# Supplementary figures and images for: Two snakebite antivenoms have potential to reduce Eswatini’s dependency upon a single, increasingly unavailable product: Results of preclinical efficacy testing
Source: PLoS Negl Trop Dis. 2022 Sep 15;16(9):e0010496. doi: 10.1371/journal.pntd.0010496 (PMC9529146; doi:10.1371/journal.pntd.0010496)

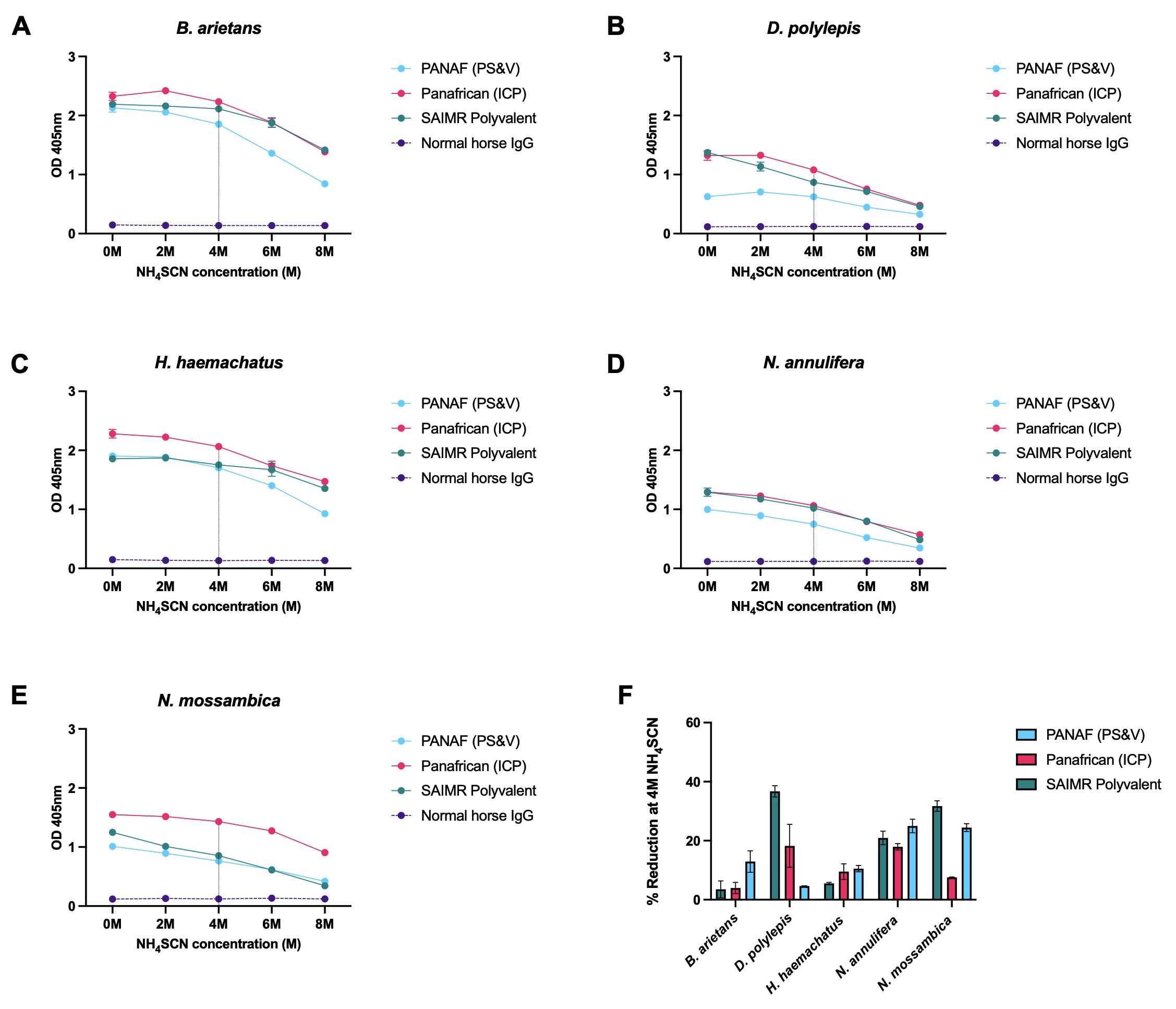

Supplement: S1 Fig — (TIFF) [file pntd.0010496.s001.tiff]

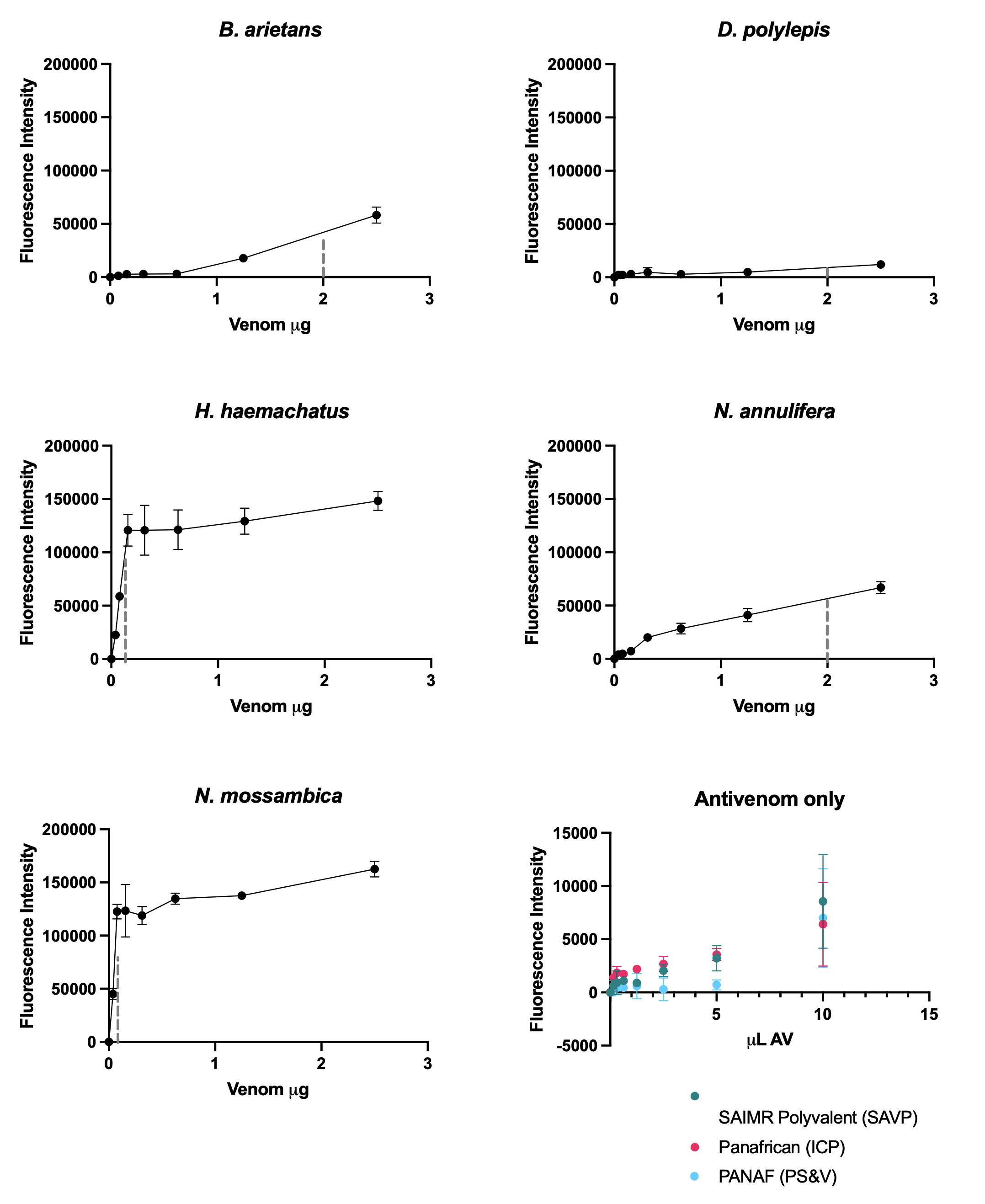

Supplement: S2 Fig — (TIFF) [file pntd.0010496.s002.tiff]

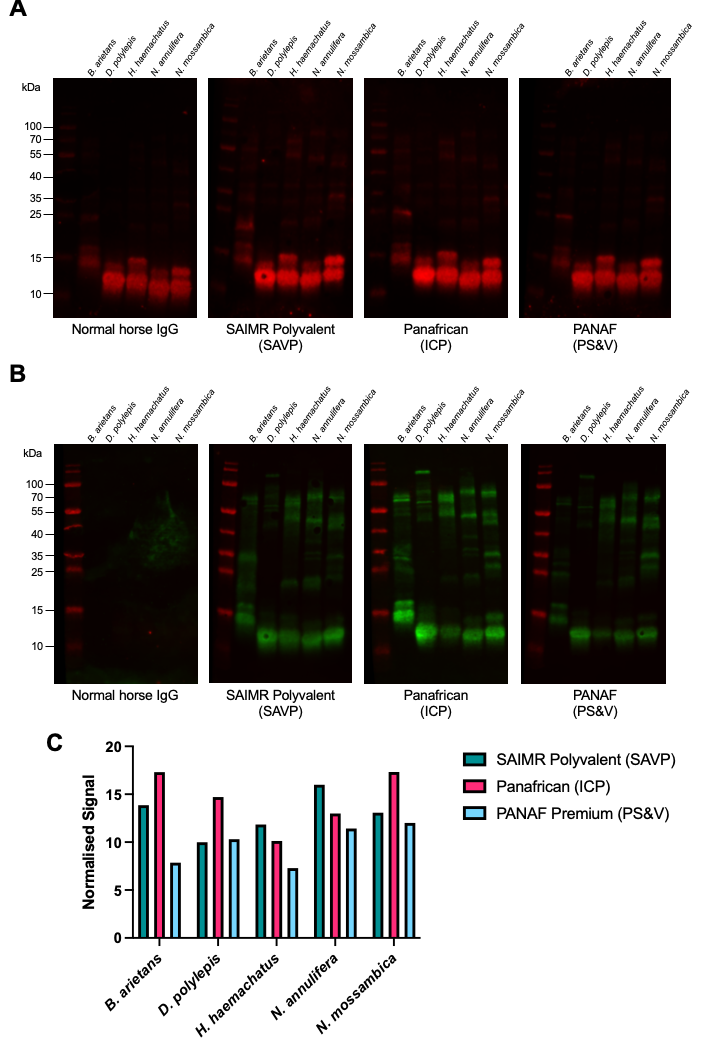

Supplement: S3 Fig — (TIFF) [file pntd.0010496.s003.tiff]
